# Identification of genomic biomarkers for anthracycline-induced cardiotoxicity in human iPSC-derived cardiomyocytes: an in vitro repeated exposure toxicity approach for safety assessment

**DOI:** 10.1007/s00204-015-1623-5

**Published:** 2015-11-04

**Authors:** Umesh Chaudhari, Harshal Nemade, Vilas Wagh, John Antonydas Gaspar, James K. Ellis, Sureshkumar Perumal Srinivasan, Dimitry Spitkovski, Filomain Nguemo, Jochem Louisse, Susanne Bremer, Jürgen Hescheler, Hector C. Keun, Jan G. Hengstler, Agapios Sachinidis

**Affiliations:** 1 Institute of Neurophysiology and Center for Molecular Medicine Cologne (CMMC), University of Cologne, Robert-Koch-Str. 39, 50931 Cologne, NRW Germany; 2 Biomolecular Medicine, Department of Surgery and Cancer, Imperial College London, London, UK; 3 Institute for Health and Consumer Protection, Joint Research Centre, European Commission, Ispra, Italy; 4 Leibniz Research Centre for Working Environment and Human Factors at the Technical University of Dortmund (IfADo), 44139 Dortmund, Germany

**Keywords:** Cardiotoxicity, Human stem cells derived cardiomyocytes, Heart failure, Transcriptomics, Genomic biomarkers, In vitro test system, Safety assessment

## Abstract

The currently available techniques for the safety evaluation of candidate drugs are usually cost-intensive and time-consuming and are often insufficient to predict human relevant cardiotoxicity. The purpose of this study was to develop an in vitro repeated exposure toxicity methodology allowing the identification of predictive genomics biomarkers of functional relevance for drug-induced cardiotoxicity in human induced pluripotent stem cell-derived cardiomyocytes (hiPSC-CMs). The hiPSC-CMs were incubated with 156 nM doxorubicin, which is a well-characterized cardiotoxicant, for 2 or 6 days followed by washout of the test compound and further incubation in compound-free culture medium until day 14 after the onset of exposure. An xCELLigence Real-Time Cell Analyser was used to monitor doxorubicin-induced cytotoxicity while also monitoring functional alterations of cardiomyocytes by counting of the beating frequency of cardiomyocytes. Unlike single exposure, repeated doxorubicin exposure resulted in long-term arrhythmic beating in hiPSC-CMs accompanied by significant cytotoxicity. Global gene expression changes were studied using microarrays and bioinformatics tools. Analysis of the transcriptomic data revealed early expression signatures of genes involved in formation of sarcomeric structures, regulation of ion homeostasis and induction of apoptosis. Eighty-four significantly deregulated genes related to cardiac functions, stress and apoptosis were validated using real-time PCR. The expression of the 84 genes was further studied by real-time PCR in hiPSC-CMs incubated with daunorubicin and mitoxantrone, further anthracycline family members that are also known to induce cardiotoxicity. A panel of 35 genes was deregulated by all three anthracycline family members and can therefore be expected to predict the cardiotoxicity of compounds acting by similar mechanisms as doxorubicin, daunorubicin or mitoxantrone. The identified gene panel can be applied in the safety assessment of novel drug candidates as well as available therapeutics to identify compounds that may cause cardiotoxicity.

**Electronic supplementary material:**

The online version of this article (doi:10.1007/s00204-015-1623-5) contains supplementary material, which is available to authorized users.

## Introduction

Drug-induced cardiotoxicity is a major safety issue and has to be considered during drug development. Various in vivo and in vitro assays have been developed to assess the adverse effect of lead compounds on cardiac functions. Due to interspecies physiological differences, these assays often do not correctly predict the actual adverse effects of drug candidates on the human heart. Moreover, traditional approaches to toxicological testing involve extensive animal studies, thus making testing costly and time-consuming. Although primate and human primary cardiomyocytes represent highly relevant cell systems, their use is limited by ethical reasons and difficult availability (Anson et al. 2011). Above all, the pharmaceutical industry is struggling with the costly withdrawal of drugs from the market due to toxic effects, often related to cardiotoxicity (Tafuri et al. 2013). Therefore, there is an urgent need for the development of a sensitive, robust and clinically relevant in vitro system with cardiomyocytes for efficacy and safety assessment.

Human embryonic stem cells (hESCs) and human induced pluripotent stem cell (hiPSC)-derived cardiomyocytes have a high physiological relevance and show typical drug-induced changes in electrophysiological properties (Caspi et al. 2009; Reppel et al. 2005; He et al. 2003). Reproducible and large-scale production of highly purified hESCs/hiPSC-derived cardiomyocytes (hESC-CMs/hiPSC-CMs) makes them an attractive source for human cardiotoxicity tests. It is expected that human cardiomyocytes will increase the predictive ability of the adverse effects of potential drugs in humans and may replace or reduce cardiac safety assessment assays based on animal-derived primary cardiomyocytes or cardiac ion channel overexpressing cell lines (Steel et al. 2009).

Among anti-cancer drugs, anthracycline family members such as doxorubicin, daunorubicin and mitoxantrone are known to induce cardiotoxicity (Menna et al. 2012; Paul et al. 2007). Multiple mechanisms such as free radical formation, lipid peroxidation and DNA damage have been proposed to explain the cardiotoxicity of anthracyclines. Additionally, interactions of anthracyclines with the DNA-topoisomerase complex or directly with DNA by intercalation result in disturbances in DNA replication and transcription and have been extensively studied (Minotti et al. 2004). Dose-dependent cardiotoxicity of anthracyclines limits their therapeutic application. Drugs that do not compromise the electrophysiology of the heart can also be cardiotoxic by directly damaging cardiomyocytes at both the subcellular and molecular levels via the formation of reactive oxygen species, DNA damage, mitochondrial damage, apoptosis or disturbed molecular signalling events. Elevated levels of cardiac troponin I (cTnI) and cardiac troponin T (cTnT) in blood correlate well with myocardial injury and act as critical plasma biomarkers for the diagnosis of cardiac damage in clinical and preclinical studies (Babuin and Jaffe 2005; O’Brien 2008; Tonomura et al. 2009). However, high levels of these biomarkers occur only after cardiac damage and can be detected for only a few hours after myocardial infarction and cardiotoxic drug treatment. To avoid drug-induced cardiotoxicity in the future, there is an urgent need to develop sensitive and reliable methods to detect or predict early cardiotoxic events.

In the present study, well-characterized hiPSC-CMs were used as an in vitro system of cardiotoxicity in combination with transcriptomics. Among the anthracyclines, doxorubicin is one of the most successful agents for solid and haematological malignancies in both children and adults. Doxorubicin has been extensively studied in a variety of preclinical models and clinical phases. Here, we developed a methodology allowing single and repeated chronic exposures of human cardiomyocytes to doxorubicin (156 nM). The cardiotoxic effects of doxorubicin were monitored by real-time counting of the beating activity and cytotoxicity. Moreover, global gene expression changes were studied using a transcriptomic approach. Doxorubicin-deregulated expression signature of genes was further analysed in a follow-up study using daunorubicin and mitoxantrone, which also belong to the anthracycline family. Our study demonstrates that the integrative use of the xCELLigence Real-Time Cell Analyser (RTCA) cardio system and toxicogenomics offers a methodology to identify cardiotoxic compounds.

## Materials and methods

### Cardiomyocyte cell culture

All experiments were performed with purified human iCell Cardiomyocytes^®^ (Cellular Dynamics International, Madison, WI, USA), which were derived from hiPSCs. The cardiomyocytes were supplied as a cryopreserved single cell suspension of a 98 % pure population. The cardiomyocytes were a mixture of spontaneously electrically active atrial-, nodal- and ventricular-like myocytes. These cells exhibit typical biochemical, electrophysiological and mechanical characteristics of normal human heart cells with expected responses upon exposure to exogenous agents. Cryopreserved hiPSC-CMs were thawed in iCell cardiomyocytes plating medium (iCell-PM, Cellular Dynamics International, Madison, WI, USA) per the manufacturer’s instructions. For functional studies, thawed cells were directly plated on a fibronectin-coated (5 µg/cm^2^, 2 h at 37 °C) E-plate Cardio 96 (ACEA Biosciences, San Diego, CA, USA) at approximately a 25 × 10^3^ cells per well density using iCell-PM. For transcriptomic studies, thawed cells were plated on fibronectin-coated (5 µg/cm^2^, 2 h at 37 °C) 6-well plates at a 0.4 × 10^6^ per well cell density. Two days later, cells were maintained in iCell cardiomyocyte Maintenance Medium (iCell-MM, Cellular Dynamics International, Madison, WI, USA), with a fresh medium change after every 2 days. The cardiomyocytes were cultured in a standard cell culture incubator at 5 % CO_2_, 37 °C.

### Chemical compounds

The 10 mM stock solutions (in DMSO) of doxorubicin, daunorubicin and mitoxantrone were purchased from Selleck Chemicals. Stock solutions were stored as small volume aliquots in tightly sealed sterile tubes at −80 °C. Drug dilutions were performed in pre-warmed (37 °C) iCell-MM prior to each drug exposure. Doxorubicin was used as the gold standard reference compound to develop experimental methodology.

### xCELLigence RTCA Cardio system

The xCELLigence RTCA Cardio system (ACEA Biosciences, San Diego, CA, USA) is an impedance-based platform for monitoring the real-time beating function of cardiomyocytes. It was used to sensitively and quantitatively detect pro-arrhythmic drug effects on cardiac function and to measure cell viability in real time. Impedance measurements were monitored at regular time intervals. The amount of growth area covered in an E-plate Cardio 96 due to cell adhesion was represented as the Cell Index (CI). A high CI indicates more cell adhesion. Before cell plating, the background impedance of E-plate Cardio 96 (ACEA Biosciences, San Diego, CA, USA) was measured using iCell-PM (50 µl per well). The raw data of cell viability, beating activity and the beating amplitude were acquired using the xCELLigence RTCA Cardio system and analysed using RTCA Cardio software version 1.0 (ACEA Biosciences, Inc, San Diego, CA, USA).

### RNA extraction

Cell samples were homogenised with QIAzol lysis reagent (Qiagen, Hilden, Germany), and the total RNA was extracted and purified using the RNeasy mini kit (Qiagen, Hilden, Germany) according to the manufacturer’s instructions. A Nanodrop (ND-1000, Thermo Fisher, Langenselbold, Germany) was used for RNA quantification and purity assessment. RNA integrity was confirmed using the Experion™ automated electrophoresis system (Bio-Rad, Munich, Germany). Extracted RNA was subjected to human gene array processing using Affymetrix’s kits, reagents and instrument setup.

### Microarray labelling and hybridization

For microarray gene expression studies, 100 ng of total RNA was used as a starting material. The total RNA samples were amplified and labelled using GeneChip 3′ IVT Express Kit per the manufacturer’s instructions (Affymetrix, High Wycombe, UK). The amplified biotin-labelled RNA (aRNA) samples were purified using magnetic beads, and 15 µg of aRNA was fragmented with fragmentation buffer per the manufacturer’s instructions. Then, 12.5 µg of fragmented aRNA was used to hybridize with Affymetrix Human Genome U133 plus 2.0 array along with the hybridization cocktail solution. For microarray hybridization, gene chips were placed in a GeneChip Hybridization Oven-645 (Affymetrix, High Wycombe, UK) for 16 h at 60 rpm and 45 °C. After incubation, the arrays were washed and stained using the Affymetrix HSW kit on GeneChip Fluidics Station-450. The stained arrays were scanned with Affymetrix GeneChip Scanner-3000-7G, and image and quality control assessments were performed with Affymetrix GCOS software. The generated CEL files were used for further statistical analysis.

### Microarray statistical data analysis and functional annotation analysis

Array raw data were quantile normalized using the RMA implementation of the R Affy package (Gautier et al. 2004). Differential expression was determined by the linear model implementation of the R Limma package (Minotti et al. 2004) followed by a Benjamini Hochberg multiple testing correction (1 % FDR). To specifically determine the perturbed transcripts, the expression level of transcripts in the doxorubicin (156 nM)-exposed cell samples was pairwise compared with that of day 2 and day 6 control cell samples, while doxorubicin washout cell sample transcripts were compared with day 14 control cell sample transcripts. The size of change was stated with a threshold value of fold change 2 in absolute scale. Choosing only significantly expressed probe sets, k-means cluster analysis was performed after transcript-wise normalization of signal values to a mean of 0 and an SD of 1 using Euclidean distance measurement and *k* = 6, using the Cluster 3.0 tool from the Eisen laboratory (Eisen et al. 1999). To further investigate biological functions and the pathway involvement of genes, Database for Annotation, Visualization and Integrated Discovery (DAVID) was used for functional annotation and gene ontology (GO) clustering (Dennis et al. 2003). The GeneCards database was also used to investigate annotative information about genes and its relation to human cardiac disorders (Safran et al. 2010).

### mRNA expression analysis using RT^2^ profiler PCR arrays and real-time PCR

Using 300–500 ng of total RNA, a genomic DNA elimination step and cDNA synthesis were performed with the RT^2^ First Strand kit (Qiagen, Hilden, Germany) according to the manufacturer’s instructions. For quantitative comparison of mRNA levels, real-time PCR was performed using custom made RT^2^ Profiler PCR array (96-well plate) (Qiagen, Hilden, Germany). This array contained 84 target genes, 5 housekeeping genes, 1 genomic DNA control, 3 reverse transcription controls and 3 positive PCR controls. Real-time PCR was performed using RT^2^ SYBR^®^ Green ROX^™^ qPCR master mix in an Applied Biosystems 7500 FAST Real-Time PCR System in accordance with the manufacturer’s recommended thermal cycling conditions. The relative gene expression analysis was performed using the 2^−ΔΔCt^ method with the RT^2^ PCR array data analysis online tool. Expression data were normalized using the geometric mean of 5 housekeeping genes—*ACTB*, *B2M*, *GAPDH*, *HPRT1* and *RPLP0*. A cut-off fold change value of 1.9 was set for significantly deregulated genes and later used to generate the gene list used for Venn diagram analysis.

### Immunostaining

For immunocytochemistry analysis, control, doxorubicin-exposed and washout iPSC-CMs were fixed with ice-cold 99 % methanol (Roth, Karlsruhe, Germany) for 10 min at −20 °C. Then, cells were permeabilized with 0.3 % Triton X-100 (Sigma-Aldrich, Steinheim, Germany) for 20 min at room temperature. Cells were blocked with 5 % bovine serum albumin (Sigma, Steinheim, Germany) for 1 h at room temperature and incubated with anti-sarcomeric alpha actinin (Abcam, 1:200) and anti-cardiac troponin T (Abcam, 1:200) for 1 h at 37 °C. The cells were washed 3 times with phosphate-buffered saline (PBS) with Ca^2+^ and Mg^2+^ for 5 min. Primary antibodies were detected using species matched respective Alexa Fluor-488/568-conjugated secondary antibodies (Invitrogen, Darmstadt, Germany) with 1 h incubation at 37 °C. The cells were washed 3 times with PBS for 5 min and then mounted with Prolong® Gold anti-fade mount with DAPI (Invitrogen, Darmstadt, Germany). Cell images were taken with an Axiovert 200 fluorescence microscope and Axiovision 4.3 software (Carl Zeiss).

## Results

### Experimental setup for single and repeated exposure

**Fig. 1:**
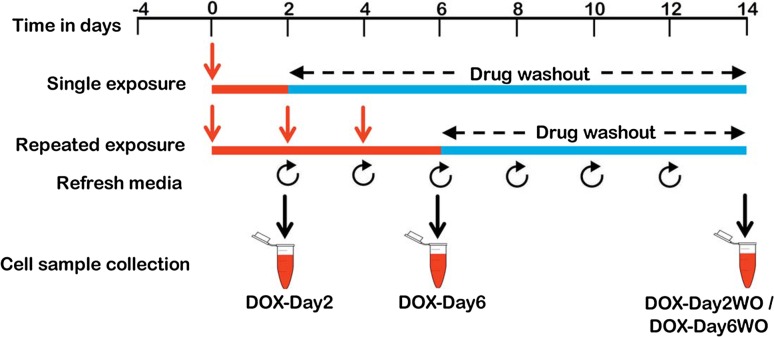
Schematic representation and experimental setup of the in vitro cardiotoxicity test system. Four days post-cell plating, the synchronously beating hiPSC-CMs were exposed to drug/test compound either for a single 2-day exposure period (single exposure) or for 6 days, consisting of 3 culture medium changes every 2 days (repeated exposure). After exposure, the drug/test compound was washed out and the cells were further incubated until day 14. For functional studies, hiPSC-CMs were seeded in the E-plate Cardio 96 and the influence of drug/test compound on cardiomyocytes was monitored by the xCELLigence RTCA Cardio system. For transcriptomics studies, RNA of drug/test compound exposed cells was harvested at day 2 and day 6, and after washout at day 2 and day 6, RNA samples were collected at day 14. Control cellular RNA samples were also harvested at the corresponding time points at day 2, 6 and 14

Cryopreserved hiPSC-CMs were thawed, and after 4 days of cultivation, synchronously beating cardiomyocytes were exposed to doxorubicin according to the timeline schematically represented in Fig. 1. In brief, an experimental setup with single 2-day exposure periods to doxorubicin (156 nM, DOX-Day2) or three consecutive exposure periods to doxorubicin (DOX-Day6) (doxorubicin supplemental media refreshed every 48 h) was applied. Until day 6, control cells were cultured in iCell-MM without doxorubicin but with DMSO as a solvent. Thereafter, cardiomyocytes were further cultivated in doxorubicin-free iCell-MM till day 14 after the start of drug exposure. Culture medium changes were performed every 2 days. For the functional studies, beating and viability data from doxorubicin-exposed cardiomyocytes were obtained by the xCELLigence RTCA Cardio system. For transcriptomic studies, doxorubicin-exposed cardiomyocytes were harvested on day 2 and day 6, respectively, whereas day 2 washout (DOX-Day2WO) and day 6 washout (DOX-Day6WO) cells were harvested on day 14. RNA isolated from the harvested cells was further analysed by gene array. The time frame from day 4 and day 18 was chosen for the present study because hiPSC-CMs (after cell plating) can be cultured in an E-plate cardio 96 until day 18–19 without losing cell viability. Moreover, the cells showed synchronous beating behaviour from day 4 onwards. In addition, the initial concentration-dependent cytotoxicity studies demonstrated that doxorubicin at 156 nM was still in a range that causes <10 % cell death, and its influence on beating rate was also less than 30 %. In contrast, doxorubicin concentrations higher than 156 nM increased cytotoxicity and arrhythmic beating in hiPSC-CMs in a concentration-dependent manner (Supplementary Figs. S1 and S2).

### Repeated exposure to doxorubicin induced arrhythmic beats in cardiomyocytes

**Fig. 2:**
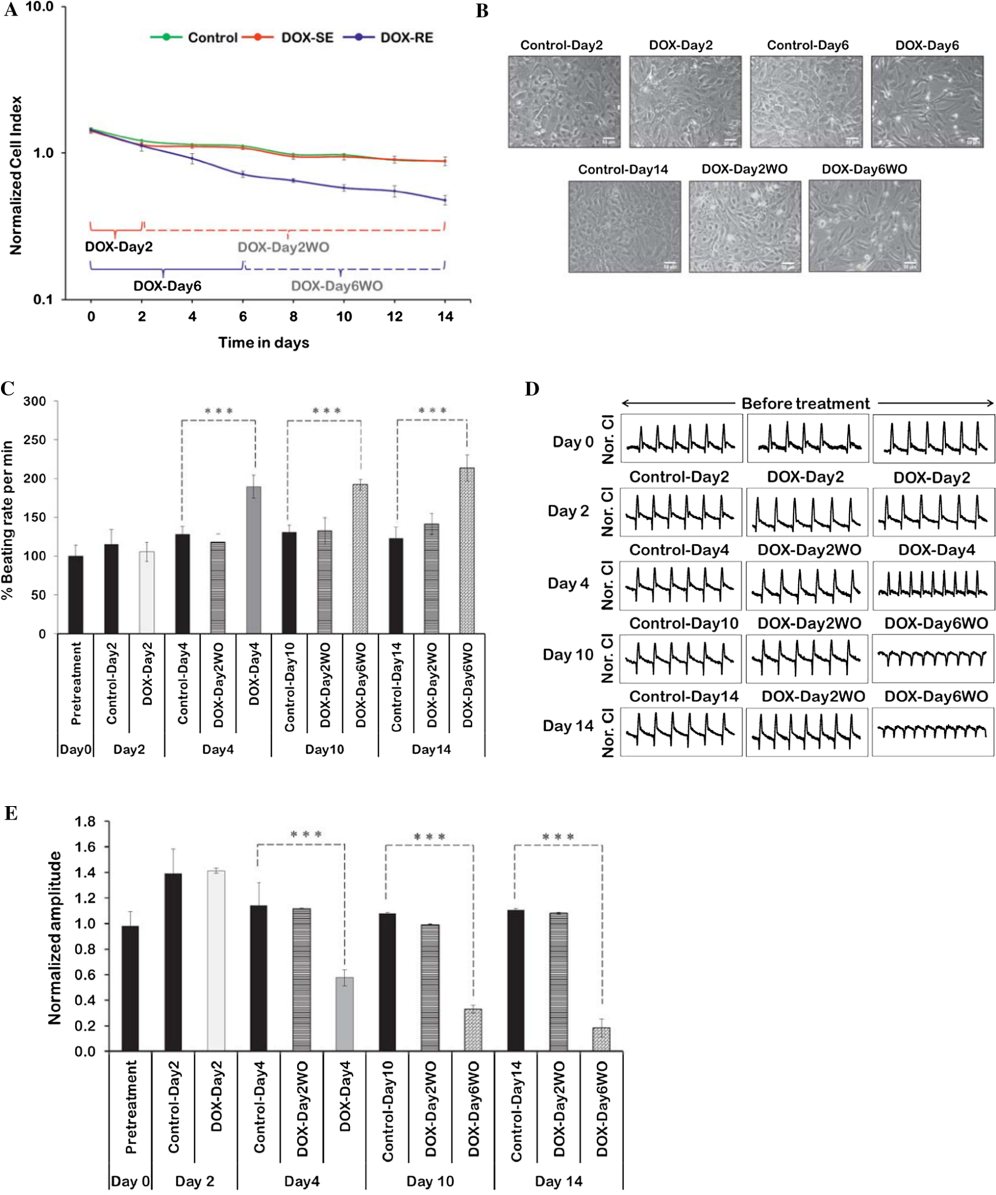
Functional studies of doxorubicin-exposed hiPSC-CMs using the xCELLigence RTCA Cardio system. **a** The representative graph displays doxorubicin repeated exposure induced cytotoxicity showing a decrease in normalized Cell Index values. In the *graph*, DOX-SE indicates doxorubicin single exposure and DOX-RE represents doxorubicin repeated exposure. Cell Index normalization was performed at a Cell Index value of 7.0. The numerical data represent means ± standard deviations (SD) (*n* = 3). **b** Influence of single and repeated doxorubicin exposures (156 nM) on cell density and morphology. The *scale bar* represents 50 µm. **c** Doxorubicin single and repeated exposures induced changes in % beating rates. Basal beating rate of cardiomyocytes was from 25 to 32 per min at threshold 10. Data show the mean ± SD (*n* = 3). ***Indicates *p* value <0.005. **d** Representative 12 s beating traces of hiPSC-CMs after doxorubicin single and repeated exposures and during drug washout. In *each graph* *Y*-axis represents normalized Cell Index (Nor. CI). Beating activity illustrates the development of arrhythmic beating upon repeated exposure and also in surviving cells after repeated exposure. **e** In contrast to single exposure, repeatedly exposed cells showed a significant decrease in beating amplitude when compared to control cells. *Bar graph* numerical values represent mean ± SD (*n* = 3). ***Indicates *p* value <0.005

Repeated doxorubicin exposure induced a decrease in normalized Cell Index values, which indicates a decrease in cell viability (Fig. 2a). During drug washout, especially in repeatedly exposed cells, a decrease in Cell Index values was observed but at a slow rate. Repeated doxorubicin exposure induced cytotoxicity with a loss of cells in the doxorubicin DOX-Day6 and DOX-Day6WO groups (Fig. 2b). Analysis of data on beating cardiomyocytes showed that repeated doxorubicin exposure increased the beating rate at day 4 (DOX-Day4), and the same effect was still observed after long-term drug washout (Fig. 2c), whereas repeated exposure at day 6 decreased the beating rate (data not shown). This may be due to the cytotoxic effect of doxorubicin on cardiomyocytes under these exposure conditions. Compared to controls, doxorubicin single exposure did not influence beating rates significantly during exposure and washout. Representative changes in beating activity were captured after doxorubicin exposure and washout (Fig. 2d). Arrhythmic beating activity was observed in DOX-Day6 and DOX-Day6WO cells. Unlike DOX-Day2 cells, repeated exposure caused a significant decrease in the beating amplitude during exposure and washout (Fig. 2e). This may be explained by the decreased number of cells contributing to the total contraction.

### Genome-wide analysis of cells with and without doxorubicin washout identifies clusters of reversibly and irreversibly altered genes

**Fig. 3:**
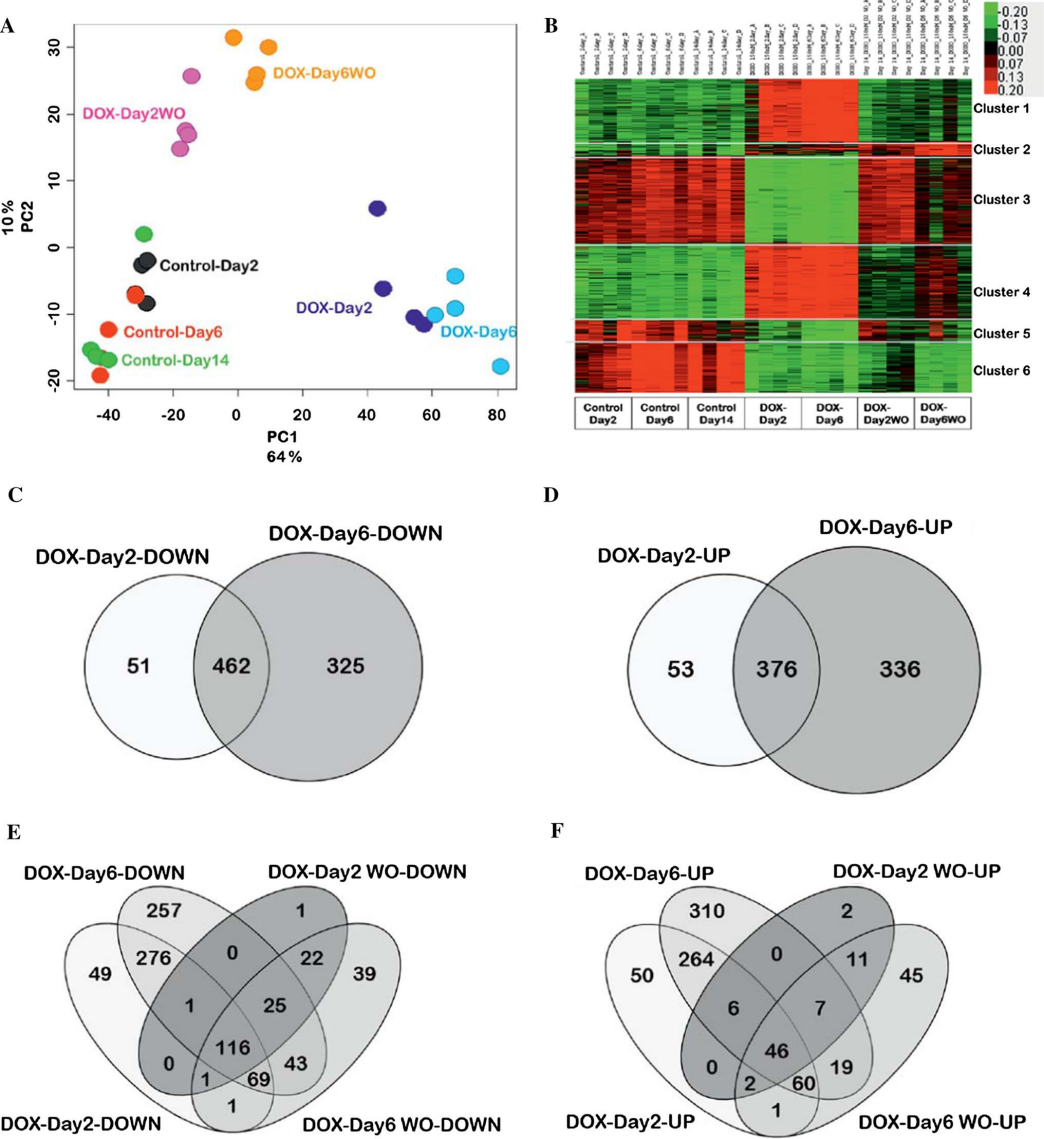
Doxorubicin-induced differential gene regulation in cardiomyocytes. **a** 2D Principal component analysis (PCA) showed that doxorubicin exposure induces changes in gene expression depending on the exposure period. Each of the four replicates obtained from independent experiments is shown in *single-coloured solid spheres*. **b** k-means clustering analysis of 2195 transcripts differentially expressed between day 2, 6 and 14. Differentially expressed transcripts were grouped into six clusters based on their expression pattern upon doxorubicin exposure and washout (**a**, **b** FDR *p* value <0.05; fold change ≥2.0). **c**, **d** Venn diagrams illustrating overlapping down- and up-regulated genes between DOX-Day2 and DOX-Day6 groups. **e**, **f** Venn diagrams representing long-term down- and up-regulated genes amongst all four experimental groups—DOX-Day2, DOX-Day6, DOX-Day2WO and DOX-Day6WO

To obtain an overview of genome-wide gene expression alterations induced by the different exposure schedules to doxorubicin, principal component analysis (PCA) was performed based on the significantly altered transcripts (FDR *p* value <0.05; fold change ≥2.0) (Fig. 3a). The first component PC1 described 64 % of the variance of the data and showed clear separation between controls and doxorubicin-exposed and washout samples. The second component (PC2) described 10 % of the variance within the data and represented the distance between samples with and without washout. During the drug washout period, DOX-Day2WO returned almost to control levels, whereas DOX-Day6WO showed a much smaller degree of recovery. Genes with at least twofold changes in expression values were identified and analysed by k-means clustering (Fig. 3b). This technique identified clusters of genes with different response patterns to doxorubicin and also distinct reversibility after washout of the test compound. Cluster 1 genes showed up-regulation after doxorubicin exposure, but returned to basal levels after drug washout (Fig. 3b). The strongest overrepresented GO terms in this cluster represented genes involved in nucleosome organization and DNA-protein complex assembly (Table 1). Cluster 2 genes were up-regulated by doxorubicin exposure and interestingly remained up-regulated even after washout of the test compound (Fig. 3b). In this cluster, endoplasmic reticulum genes and the endoplasmic reticulum-nuclear signalling pathway were overrepresented. Genes in cluster 3 were down-regulated by doxorubicin and mostly recovered after the washout (Fig. 3b). Interestingly, the strongest overrepresented GO groups in this cluster were associated with the sarcomere, myofibrils, contractile fibre part and regulation of heart contraction. Therefore, cluster 3 contains genes that were clearly associated with normal heart function and therefore should be of high interest as biomarkers of cardiotoxicity. Expression of cluster 4 genes was up-regulated upon doxorubicin exposure and decreased again after washout of the test compound, although not completely to control levels. Cluster 4 genes represented a cell stress response, with the p53 signalling pathway and apoptosis GO terms being overrepresented. Cluster 5 was a relatively small group of genes that were down-regulated after doxorubicin exposure and included overrepresented endogenous metabolism and extracellular matrix genes. Cluster 6 genes were down-regulated by doxorubicin and remained repressed even after washout. A strong overrepresentation of mitosis-associated genes occurred in this cluster. The genes of the individual clusters are listed in Supplemental Table 1, and all significant GO groups of the clusters are provided in Supplemental Table 2.

**Table 1:** Overrepresented GO categories and pathways of k-means cluster genes

Term	Gene count	*p* value
Cluster 1		
GO:0000786—nucleosome	12	3.78E−09
GO:0006334—nucleosome assembly	13	7.54E−09
GO:0065004—protein-DNA complex assembly	13	1.91E−08
Cluster 2		
GO:0005783—endoplasmic reticulum	14	8.80E−05
GO:0006984—ER-nuclear signalling pathway	4	2.76E−04
GO:0005509—calcium ion binding	12	4.54E−04
Cluster 3		
GO:0030017—sarcomere	17	2.04E−09
GO:0030016—myofibril	17	1.31E−08
GO:0044449—contractile fibre part	17	1.70E−08
GO:0008016—regulation of heart contraction	14	3.25E−08
Cluster 4		
hsa04115:p53 signalling pathway	16	9.09E−11
GO:0043067—regulation of programmed cell death	45	1.80E−08
GO:0042981—regulation of apoptosis	44	3.88E−08
Cluster 5		
GO:0019752—carboxylic acid metabolic process	18	1.04E−06
GO:0006520—cellular amino acid metabolic process	11	9.33E−06
GO:0005583—fibrillar collagen	4	6.40E−05
Cluster 6		
GO:0000279—M phase	78	6.83E−66
GO:0007067—mitosis	61	5.46E−55
GO:0000087—M phase of mitotic cell cycle	61	1.82E−54

### Doxorubicin exposure down-regulates genes of cardiac function and up-regulates stress-associated genes

Doxorubicin exposure led to 2195 differentially expressed probe sets (FDR *p* value <0.05; fold change ≥2), which were used for further analysis. Venn diagram analysis of deregulated genes between DOX-Day2 and DOX-Day6 displayed a list of 462 mutually down- and 376 mutually up-regulated genes (Fig. 3c, d). These commonly down- and up-regulated genes were used separately for GO enrichment analysis using the DAVID functional enrichment tool. This analysis led to the identification of early doxorubicin-responsive cardiac processes, pathways and general toxic responses. The GO analysis showed that down-regulated genes were mainly enriched in GOs such as muscle contraction, sarcomere, cytoskeleton and 6 KEGG pathways (Table 2), whereas up-regulated GOs were mainly enriched in cell death, anti-apoptosis, DNA damage stimulus, oxidative stress responses and the KEGG pathways such as the p53 signalling pathway and apoptosis (Table 3).

**Table 2:** Significantly enriched GO categories and pathways by commonly down-regulated genes between DOX-Day2 and DOX-Day6

Components	Gene count	*p* value	Representative genes
GO term			
GO:0006936—muscle contraction	23	3.4E−11	*SLC8A1, TCAP, ACTA1, MYL3, PGAM2, MYH7, MYH6, TNNI3, GJA5, EDNRA, DES, TNNT1, MYOM2, ARG2, RYR2, ASPH, KCNH2, SCN5A, KCNQ1, CASQ2, HRC, SGCA, MB*
GO:0005856—cytoskeleton	74	1.9E−10	*KIF23, PRC1, TTK, AURKA, AURKB, GTSE1, KIF2C, FRMD5, DES, ANK2, LMOD2, MYC, TOP2A, LMOD3, TUBA1B, ASPM, KIF14, CDC6, CDK1, KIF11, ACTA1, KIF15, TPX2, LDB3, NUSAP1, MYH7, MYH6, MCM3, TNNT1*
GO:0030017—sarcomere	14	8.5E−07	*MYL2, ACTA1, TCAP, MYL3, LDB3, FHL2, MYH7, MYH6, TNNI3, DES, TNNT1, ANK2, DMD, RYR2*
GO:0003013—circulatory system process	13	0.00262	*MYL2, CORIN, TCAP, MYL3, MYH6, NPR3, ATP1A2, TNNI3, EDNRA, RYR2, KCNH2, SCN5A, KCNQ1*
GO:0016529—sarcoplasmic reticulum	5	0.01542	*PYGM, SRL, RYR2, CASQ2, HRC*
KEGG pathways			
hsa04260:Cardiac muscle contraction	11	1.98E−05	*SLC8A1, MYL2, MYL3, ATP1B4, COX6A2, RYR2, MYH7, ATP1A2, MYH6, TNNI3, CACNA2D2*
hsa05410:Hypertrophic cardiomyopathy (HCM)	11	4.24E−05	*SLC8A1, DES, MYL2, MYL3, DMD, RYR2, MYH7, MYH6, TNNI3, CACNA2D2, SGCA*
hsa05414:Dilated cardiomyopathy	11	8.42E−05	*SLC8A1, DES, MYL2, MYL3, DMD, RYR2, MYH7, MYH6, TNNI3, CACNA2D2, SGCA*
hsa00240:Pyrimidine metabolism	8	0.009192	*PRIM1, NME4, NME2, NME3, NME1*-*NME2, POLE2, RRM2, POLA1, TK1*
hsa05412:Arrhythmogenic right ventricular cardiomyopathy (ARVC)	7	0.011314	*SLC8A1, DES, DMD, RYR2, CACNA2D2, SGCA, CTNNA3*
hsa00230:Purine metabolism	10	0.013765	*PRIM1, NME4, ADSSL1, NME2, NME3, NME1*-*NME2, POLE2, PDE1C, RRM2, POLA1, PAICS*

**Table 3:** Significantly enriched GO categories and pathways by commonly up-regulated genes between DOX-Day2 and DOX-Day6

Components	Gene count	*p* value	Representative genes
GO terms			
GO:0006333—chromatin assembly or disassembly	14	2.9E−07	*HIST1H2AB, HIST2H2AA3, HIST2H2AA4, HIST4H4, HIST1H4L, HIST1H4* *K, HIST1H2AG, HIST1H2AD, HIST1H2AE, HIST2H4A, HIST2H4B, H2BFS, HIST1H4A, HIST1H2BK, HIST1H4B, HIST1H2BI, HIST1H4E, HIST1H4F*
GO:0006974—response to DNA damage stimulus	19	8.6E−05	*XRCC4, RAD51C, POLH, ZMAT3, RPS27L, RRM2B, SESN1, RNF8, TRIAP1, CDKN1A, CASP3, XPC, BTG2, BAX, UBR5, AEN, DDB2, PCNA, GADD45A*
GO:0033554—cellular response to stress	24	1.2E−04	*GADD45A, XRCC4, RAD51C, POLH, ZMAT3, RPS27L, RRM2B, SESN1, RNF8, TRIAP1, DHRS2, GPX1, CASP3, CDKN1A, XPC, BTG2, AEN, UBR5, BAX*
GO:0008219—cell death	26	6.9E−04	*ZMAT3, GADD45A, BAX, KIT, STK17A, TP53INP1, PMAIP1, APLP1, GPX1, TRIAP1, CASP3, TNFRSF11B, TMEM173, AEN, FAS, TRAF4, PHLDA1, RNF144B, GAS1, NTN1, TNFRSF10A, TNFRSF10C, TNFRSF10B, TNFRSF10D,*
GO:0006979—response to oxidative stress	9	0.00772	*EGFR, GPX1, DHRS2, SDC1, RRM2B, NQO1, ETV5, ADA, OXR1*
GO:0050727—regulation of inflammatory response	6	0.01013	*GPX1, A2* *M, MASP1, ACE2, ITGA2, ADA*
GO:0005576—extracellular region	44	0.02673	*GDF15, ACE2,FGFR2, ADAMTS17, A2* *M, SORD, IGFBPL1, MASP1, NELL2, JAG1, KIT, LSR, ADA, APLP1, BDNF, TNFRSF11B, SERPINE2, COL27A1, FAS, NRG1, TFPI2, PCSK5, GFOD1, THBS4, EGFR, TMEFF2, FLRT2, ICAM4*
GO:0006916—anti-apoptosis	9	0.02699	*TRIAP1, GPX1, BDNF, TNFRSF10D, BAX, FAS, NRG1, ANXA4, GSTP1*
KEGG pathways			
hsa04115:p53 signalling pathway	16	5.2E−12	*ZMAT3, RRM2B, PMAIP1, SESN2, SESN1, EI24, TP53I3, PPM1D, CDKN1A, CASP3, TNFRSF10B, BAX, DDB2, MDM2, FAS, GADD45A*
hsa04210:Apoptosis	9	4.6E−04	*TNFRSF10A, CASP3, TNFRSF10C, TNFRSF10B, TNFRSF10D, BAX, ENDOD1, FAS, PRKX*

Moreover, Venn diagram analysis of deregulated genes in DOX-Day2, DOX-Day6, DOX-Day2WO and DOX-Day6WO revealed 116 down- and 46 up-regulated genes, which did not recover during the washout period and showed long-term deregulation (Fig. 3e, f). These 46 up-regulated genes had enriched GOs such as apoptosis, DNA damage, stress-responsive processes and the KEGG p53 signalling pathway (Table 4), whereas down-regulated genes did not show significant enrichment in cardiac- and toxicity-related biological processes as well as in the KEGG pathways. This analysis suggests that doxorubicin negatively affected the expression of many cardiac genes, which are essential for an intact function of cardiomyocytes.

**Table 4:** Long-term up-regulated genes enriched GO categories and pathways

Components	Gene count	*p* value	Representative genes
GO term			
GO:0006917—induction of apoptosis	5	0.0030	*TNFRSF10A, CDKN1A, ZMAT3, RRM2B, FAS*
GO:0033554—cellular response to stress	6	0.0039	*XRCC4, CDKN1A, ZMAT3, RRM2B, NEFL, ETV5*
GO:0006974—response to DNA damage stimulus	4	0.0338	*XRCC4, CDKN1A, ZMAT3, RRM2B*
KEGG pathways			
hsa04115:p53 signalling pathway	5	4.7E−05	*CDKN1A, ZMAT3, MDM2, RRM2B, FAS*

### Validation of deregulated genes by real-time PCR and follow-up by further cardiotoxic compounds

**Fig. 4:**
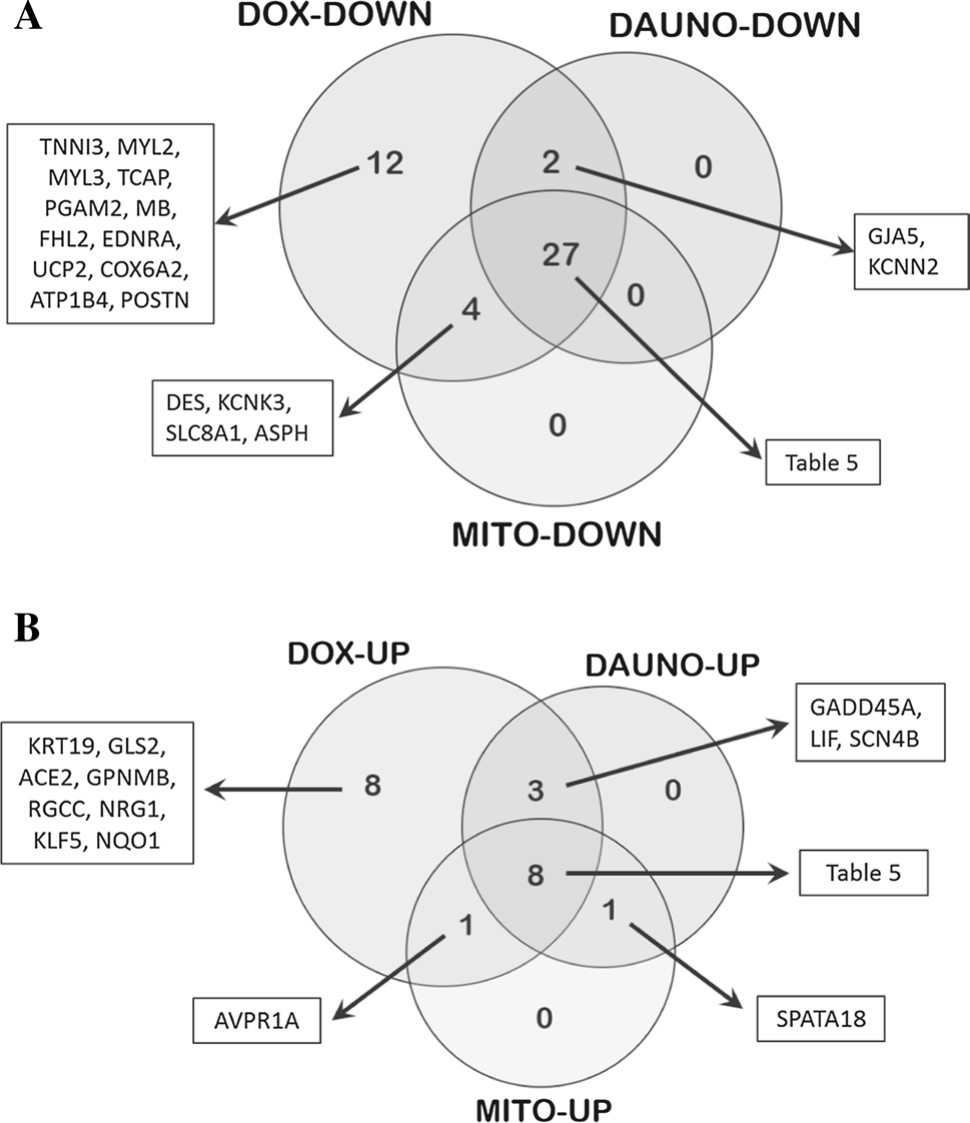
Venn diagrams representing number of common and drug-specific deregulated genes following 48 h exposure to anthracyclines in hiPSC-CMs. **a** Commonly down-regulated 27 genes and **b** commonly up-regulated 8 genes among doxorubicin (DOX), daunorubicin (DAUNO) and mitoxantrone (MITO) groups

From the above-described transcriptomic data and GO results, 84 significantly early and/or long-term deregulated genes were selected to validate their mRNA levels using real-time PCR. Most of them belong to cluster 3 (Fig. 3b), which was considered as particularly relevant because it represents contractile fibres and myofibril-associated genes. This gene panel includes 50 down- and 34 up-regulated doxorubicin-induced genes with at least a twofold change. Selected genes were mostly cardiac specific with involvement in cardiac contraction (preferably sarcomeric genes), ion homeostasis, cardiac physiology and pathophysiology, whereas another set of genes were associated with apoptosis, DNA damage and stress responses. Out of 84 genes, 63 genes were consistently deregulated upon doxorubicin single and repeated exposure (except *LIF* and *CALM1*, which were deregulated upon repeated exposure) and showed recovery during the washout period. Another 19 genes did not show full recovery during washout. Along with doxorubicin transcriptomic data validation, differential regulation of these 84 genes was also investigated in hiPSC-CMs incubated with daunorubicin (10 nM) and mitoxantrone (3 nM) using real-time PCR. The daunorubicin and mitoxantrone test concentrations were determined using dose-dependent studies in hiPSC-CMs after 48 h of drug exposure (Supplementary Fig. S1). The selected test concentrations of daunorubicin and mitoxantrone did not induce cytotoxicity by more than 10 % and also did not significantly influence beating rate or beating activity compared to controls (Supplementary Fig. S2–S4). Real-time PCR analysis revealed the deregulation of 65 genes in the doxorubicin group and confirmed the transcriptomics results by 77.4 %. The daunorubicin and mitoxantrone groups showed the deregulation of 41 genes in each group. Venn diagram analysis of the doxorubicin, daunorubicin and mitoxantrone groups displayed 35 commonly influenced genes: 27 down- and 8 up-regulated genes (Fig. 4a, b; Table 5).

**Table 5:** Gene expression studies (fold regulation) by real-time PCR in hiPSC-CMs after 48 h exposure of doxorubicin (156 nM), daunorubicin (10 nM) and mitoxantrone (3 nM)

Gene symbol	Fold regulation			*p* value		
	Doxorubicin	Daunorubicin	Mitoxantrone	Doxorubicin	Daunorubicin	Mitoxantrone
*ACTA1*	−211.2	−5.0	−11.7	7.5E−05	9.7E−03	4.6E−03
*TNNT1*	−31.1	−2.7	−2.7	3.4E−05	6.8E−03	7.3E−03
*HRC*	−467.6	−38.1	−48.1	8.2E−04	5.2E−04	2.9E−03
*MYOM2*	−50.0	−9.7	−22.1	6.2E−04	2.1E−03	9.8E−03
*MYH6*	−290.8	−60.3	−76.8	1.5E−04	3.2E−04	5.5E−04
*MYH7*	−2684.9	−182.1	−179.4	2.7E−05	6.9E−05	3.9E−04
*ANK2*	−87.1	−10.4	−3.5	2.2E−03	5.4E−03	9.1E−02
*LDB3*	−128.4	−12.0	−19.3	9.4E−05	2.4E−04	5.3E−04
*DMD*	−9.6	−10.4	−22.4	2.1E−03	4.8E−04	7.5E−04
*NRAP*	−21.8	−19.4	−22.8	9.5E−05	2.3E−04	1.5E−03
*ATP1A2*	−248.2	−9.3	−25.6	1.9E−02	2.3E−03	8.3E−03
*KCNQ1*	−29.1	−4.2	−4.9	1.3E−04	3.1E−03	8.2E−03
*KCNH2*	−29.1	−5.9	−6.3	2.6E−04	8.9E−03	1.3E−02
*SCN2B*	−19.7	−4.7	−5.1	7.6E−04	8.5E−03	1.1E−02
*SCN5A*	−119.9	−37.6	−28.2	3.1E−04	1.9E−03	1.8E−03
*CACNA2D2*	−52.4	−68.3	−59.4	8.1E−03	5.0E−03	4.2E−03
*PYGM*	−457.1	−8.2	−11.8	1.4E−04	4.4E−04	4.8E−04
*CKM*	−71.1	−2.5	−1.9	6.1E−05	2.9E−03	3.1E−02
*MURC*	−22.8	−9.6	−7.4	1.3E−03	8.3E−04	1.0E−03
*ERBB3*	−20.5	−7.3	−5.0	2.6E−04	2.9E−03	2.7E−03
*JAK2*	−18.6	−3.2	−4.4	4.9E−04	1.8E−02	1.8E−03
*PPP1R3A*	−15.2	−3.5	−7.9	5.0E−03	1.9E−03	1.0E−02
*RYR2*	−136.7	−26.9	−82.5	1.6E−03	1.3E−04	5.3E−03
*IRX4*	−21.4	−5.6	−6.2	2.4E−04	7.5E−03	3.4E−03
*CORIN*	−68.8	−5.9	−15.9	5.9E−04	3.6E−03	2.9E−03
*CACNA1G*	−75.5	−62.0	−42.4	6.5E−04	2.1E−03	1.7E−04
*PRDM16*	−35.3	−28.5	−35.8	1.3E−02	6.2E−04	9.8E−04
*BAX*	7.72	3.91	2.93	2.9E−04	3.7E−04	5.5E−04
*ZMAT3*	3.3	3.4	2.0	5.3E−03	3.4E−03	1.6E−02
*GDF15*	8.8	2.6	6.9	5.2E−04	1.5E−02	8.7E−03
*FAS*	18.3	7.3	5.7	3.1E−05	3.6E−05	4.2E−05
*PRKX*	1.9	2.6	2.1	7.3E−02	1.5E−02	1.1E−02
*DUSP4*	15.8	3.1	2.5	1.9E−03	8.7E−03	2.2E−02
*KCNJ2*	18.7	2.3	2.8	1.3E−04	1.8E−02	6.4E−03
*GPX1*	15.8	2.4	2.6	1.6E−04	7.3E−03	2.6E−03

The numerical data represent the fold regulation values compared to controls. The *p* value is calculated based on a Student’s *t* test of the Ct values (*n* = 3). *p* value ≤0.05 is considered significant

### Doxorubicin-induced sarcomeric deterioration

**Fig. 5:**
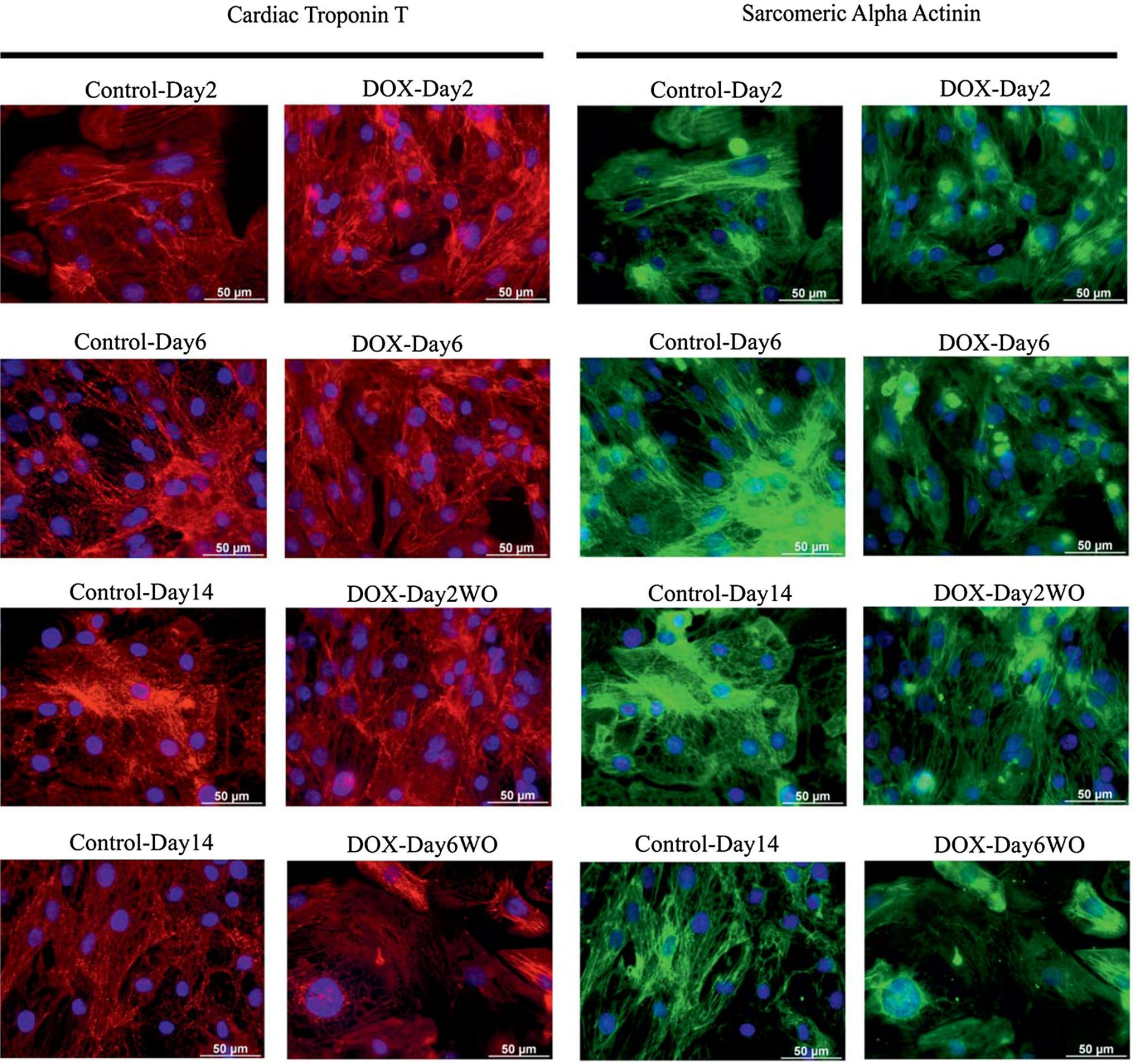
Immunohistochemistry of cardiac troponin T and sarcomeric cardiac α-actinin proteins in control, doxorubicin-exposed and washout hiPSC-CMs. *Red* and *green colours* indicate cardiac troponin T and sarcomeric alpha actinin staining, respectively. *Blue colour* indicates nuclear staining. Immunofluorescence results demonstrate a significant decrease in both protein expression levels in DOX-Day6 and DOX-Day6WO cells. *Scale bar* represents 50 µm (colour figure online)

Our results showed that doxorubicin single exposures induced the down-regulation of sarcomeric genes. To investigate the effect of doxorubicin single and repeated exposures on proteins relevant for sarcomere structure, cardiac troponin T and sarcomeric alpha actinin in cardiomyocytes were studied by immunohistochemistry. Unlike doxorubicin single exposures, repeated exposures decreased the expression of both sarcomeric proteins and resulted in an irregular structure of the troponin and actinin filaments as compared to untreated cardiomyocytes (Fig. 5).

## Discussion

For reliable evaluation of cardiotoxicity, human relevant models are urgently required. Current preclinical cardiac safety studies have mainly relied on cardiac ion channels, especially the human ether-a-go–go (hERG) channel, as well as in vivo tests. In the present study, we have established an in vitro cardiotoxicity methodology for the monitoring of early as well as chronic cardiotoxicity events at the cellular, functional and genomic level.

Doxorubicin is an established anti-cancer drug with well-known cardiac injury side effects. Its cumulative dose-dependent cardiotoxicity (Appel et al. 2007; Menna et al. 2012) leads to congestive heart failure (Haq et al. 1985; Ibrahim et al. 1999). Doxorubicin has a tendency to disturb cardiac rhythm and in some cases can cause life-threatening arrhythmia or even sudden death (Lacasse and Bolduc 1992). The doxorubicin-induced disturbances in cardiac function can be detected within a few hours or within 24–48 h following doxorubicin administration by electrocardiography in human and animal hearts (Dindogru et al. 1978; Friess et al. 1985; Kehoe et al. 1978).

Our findings demonstrate that, unlike single exposure, repeated doxorubicin exposure caused arrhythmic beating on day 4 and induced substantial cytotoxicity accompanied with decreased beating amplitude on day 6. Doxorubicin-induced arrhythmic beating indicates disturbed cardiac function, while the decreased amplitude reflects reduced contractile force. Our results are in agreement with findings demonstrating that chronic doxorubicin administration reduces contractile function in rabbit hearts (Boucek et al. 1997).

The GO and KEGG pathway analysis of our transcriptomic studies showed that doxorubicin exposure preferentially suppressed the expression of genes involved in cardiac contraction and pathways related to cardiomyopathies. In addition, doxorubicin exposure also deregulated genes with enriched biological processes such as apoptosis, DNA damage and the oxidative stress response. These observations show that in addition to a general stress response, genes involved in sarcomeric and cardiac muscle contraction are more responsive to doxorubicin exposure. Cardiomyocyte sarcomeres are highly organized structures of myofilaments and are involved in mechanical cardiac contraction. Our transcriptomic data showed significant down-regulation of sarcomere genes in DOX-Day2 and DOX-Day6 cells. Similar findings have also been reported in rat cardiomyocytes, in which chronic doxorubicin exposure induces significant degeneration of sarcomeres (Sussman et al. 1997). Our immunocytochemical analysis showed that compared to control and DOX-Day2 cells, repeated doxorubicin exposure resulted in decreased expression of cardiac troponin T and sarcomeric α-actinin proteins in DOX-Day6 cells, and this expression remained at lower levels even after doxorubicin washout in DOX-Day6WO. In addition, disorganization of myofibrillar structures in the DOX-Day6 cardiomyocytes has been observed. Doxorubicin-induced myofibrillar disarray has also been reported in rat ventricular cardiomyocytes (Sussman et al. 1997; Sawyer et al. 2002).

Regulation of ion homeostasis is one of the essential functional elements during cardiac contraction. Intracellular calcium (Ca^2+^) is the central regulator of cardiac contraction, and its homeostasis is tightly regulated by Ca^2+^ ion channels, Ca^+2^ receptors and Ca^+2^ binding proteins. Our data showed down-regulation of Ca^2+^-transporting genes such as *RYR2*, *SLC8A1*, *CACNA1G*, *CACNA2D2* and *ITPR1* in doxorubicin-exposed hiPSC-CMs. Differential regulation of two calcium release channels (RYR2 and ITPR1) has been reported during end stage heart failure (Go et al. 1995). Chronic rabbit heart studies have shown alterations in Ca^2+^ release causing abnormalities of contractions and relaxation in doxorubicin-induced cardiomyopathy (Dodd et al. 1993). In addition, we found down-regulation of sodium (Na^+^) and potassium (K^+^) ion channel encoding genes, such as *KCNQ1*, *KCNK3*, *KCNN2*, *KCNH2*, *SCN2B* and *SCN5A*. Deregulated Na^+^ and K^+^ ion channels play an important role in cardiac arrhythmias and heart failure (Remme and Bezzina 2010; Nabauer and Kaab 1998). In summary, our results are in accordance with animal and clinical studies demonstrating that doxorubicin induces disturbances in cardiac calcium homeostasis as well as altered sodium and potassium ion channel activity. In the present study, doxorubicin exposure significantly deregulated ion homeostasis maintaining genes at the mRNA level accompanied with functional changes in the beating behaviour of hiPSC-CMs.

Mitochondrial dysfunction has been suggested to be involved in doxorubicin-induced cardiotoxicity. However, the exact mechanisms of the suppressive effects by doxorubicin on the mitochondrial electron transport chain, oxidative metabolism and ATP synthesis are not fully understood. As a crucial component of the mitochondrial electron transport chain, cytochrome C oxidase (CCo) and uncoupling protein (UCP) activity influences mitochondrial function at the ATP level. In rat hearts, doxorubicin treatment reduced CCo subunit expression (Chandran et al. 2009) and also down-regulated *Ucp2* and *Ucp3* expression (Bugger et al. 2011). Down-regulation of UCPs and CCo genes showed an inverse relationship with increased oxidative stress (Akhmedov et al. 2015; Bugger et al. 2011; Srinivasan and Avadhani 2012); CCo dysfunction also has a direct effect on cellular ATP levels. In agreement with these observations, our data also indicated a down-regulation of *UCP2* and *CCo* (Cytochrome C Oxidase Subunit VIa Polypeptide 2) expression, whereas up-regulation was observed for oxidative stress-responsive genes such as *NQO1*, *OXR*, *GCH1* and *GPX1* in doxorubicin-exposed hiPSC-CMs (supplementary Fig. S5). Similarly to our findings, the reduced activity of creatine kinase muscle (CKM) and myoglobin (MB) has been reported in human failing hearts (Braunlin et al. 1986; Nascimben et al. 1996; Obrien et al. 1992). Decreased levels of CKM impair the ATP delivery process to energy-consuming systems, and decreased levels of MB disturb oxygen diffusion and the mechanical functions of the cardiac muscle. In conclusion, the present results suggest that doxorubicin induces increased oxidative stress and impairs mitochondrial ATP synthesis and delivery in cardiomyocytes. Overall, these intracellular mechanisms contribute to the impaired cardiac function observed after doxorubicin treatment in cancer patients.

In accordance with our findings, the expression level of apoptosis genes including *BAX* and *FAS* has also been found to be up-regulated in human failing hearts (Latif et al. 2000; Sheppard et al. 2005). Increased expression of *BAX* and *FAS* may induce apoptosis and reduce the chances of myocardial recovery. Similarly to our results, increased expression levels of *ACE2*, *NRG1*, *DUSP4* and *LIF* have also been found in human heart failure (Goulter et al. 2004; Yan and Morgan 2011; Communal et al. 2002; Eiken et al. 2001). *KCNJ2* is also up-regulated in human dilated cardiomyopathy (Szuts et al. 2013). Notably, *GDF15* and *GPNMB* (patent publication number—WO2012072752 A1) have been proposed as diagnostic biomarkers of heart failure (Wang et al. 2010; Kempf and Wollert 2009; Khan et al. 2009) and were also up-regulated in our model system. Therefore, the gene expression responses observed in our established in vitro system showed a high degree of similarity to those of the human heart in vivo.

The Venn diagram analysis of differentially expressed genes in cells exposed to doxorubicin, daunorubicin and mitoxantrone exhibited an overlap of 27 down- and 8 up-regulated genes. The 27 down-regulated genes are mainly involved in sarcomere structure and the regulation of ion homeostasis. Up-regulated genes mainly indicate a general stress response, and they included stress markers such as *BAX*, *FAS*, *GPX1* and *ZMAT3*. This observation may help to better understand cellular mechanisms underlying late apoptosis inducing cardiac cell loss many years after anthracycline treatment. Although GDF15 has been reported to represent a biomarker for heart failure, elevated levels have also been found in the cell systems of liver, lung and kidney injury (Hsiao et al. 2000; Zimmers et al. 2005). Thus, it can be interpreted as a marker which indicates cell injury in multiple tissues. The identified 35 genes in the overlap of all three anthracyclines represent an anthracycline-responsive gene consensus expression signature and could be applied as a predictive toxicity signature for potential cardiotoxicants that act by similar mechanisms as doxorubicin, daunorubicin or mitoxantrone.

In the present study, a concentration of 156 nM doxorubicin was chosen because in the hiPSC-CMs this concentration compromises the contractility without causing major cytotoxic effects. At doses of bolus administration of doxorubicin varying between 15 and 90 mg/m^2^, the initial maximal plasma concentrations in patients are approximately 5 µM (Gewirtz 1999). After this initial peak, the plasma concentration of doxorubicin decreases rapidly, to the range of 25–250 nM, within 1 h. Similar plasma concentrations have also been reported in patients receiving continuous infusions of doxorubicin (Gewirtz 1999). In addition, after a 50 mg/m^2^ intravenous injection in adult acute myeloid leukaemia patients, a daunorubicin peak plasma concentration range from 120 to 560 nM on day 1 and 8 to 610 nM on day 3 has been observed (Lofgren et al. 2007). The mitoxantrone peak plasma concentrations have been reported to vary from 0.46 to 2.49 µM after 1 h infusion of mitoxantrone (12 mg/m^2^) and then decrease rapidly to around 10 nM within 5 h (Sundman-Engberg et al. 1993). Therefore, the concentration chosen in the present study is within the therapeutic range of anthracyclines therapy.

In summary, the results obtained by anthracyclines in hiPSC-CMs recapitulated the disturbed cardiac function observed in vivo and in clinical studies. Doxorubicin-induced adverse effects on cardiac function can be detected much earlier at the genomic level before cytotoxicity and arrhythmia can be observed. The combined application of hiPSC-CMs, the xCELLigence RTCA Cardio system and transcriptomics resulted in the identification of an anthracycline consensus signature representing early biological processes that significantly contribute to better understanding of the cardiotoxic effects of compounds both at a cellular and molecular level. The present methodology can allow for first-line in vitro preclinical tests and reduce animal usage in drug safety studies and the costs of safety evaluation. Although it is very likely that the methodology possesses a low false negative rate for severely cardiotoxic compounds, however, whether this approach can avoid false negatives for mildly cardiotoxic compounds and false positives for non-cardiotoxic compounds should be demonstrated by screening of several non- and mild cardiotoxicants.

## Electronic supplementary material

Below is the link to the electronic supplementary material.

Supplementary material 1 (DOCX 1485 kb)

Supplementary material 2 (XLSX 185 kb)

Supplementary material 3 (XLSX 81 kb)
