# Supplementary material for: Identification of genomic biomarkers for anthracycline-induced cardiotoxicity in human iPSC-derived cardiomyocytes: an in vitro repeated exposure toxicity approach for safety assessment
Source: Arch Toxicol. 2015 Nov 4;90(11):2763–77. doi: 10.1007/s00204-015-1623-5 (PMC5065579; doi:10.1007/s00204-015-1623-5)

**Supplementary figures**

**Supplementary fig. S1: C**ell viability assessment after anthracyclines treatment. In E-plate Cardio 96, synchronously beating hiPSC-CMs were treated with range of concentrations of **A.** Doxorubicin (DOX), **B.** Daunorubicin (DAUNO) and **C.** Mitoxantrone (MITO) for 48 h. Quantitative percent cell viability data showed that the concentrations like 156 nM (DOX), 10 nM (DAUNO) and 3 nM (MITO) are minimal toxic doses (less than 10% cell death) for hiPSC-CMs. In the graphs, white hollow ball points represent drug concentrations, while dark solid ball points represent minimal toxic concentrations. Error bars displays mean ± Standard Deviation.

**A. B. C.**


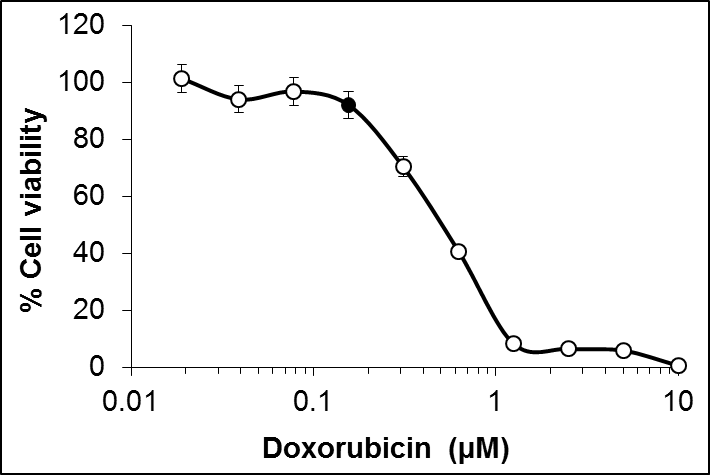

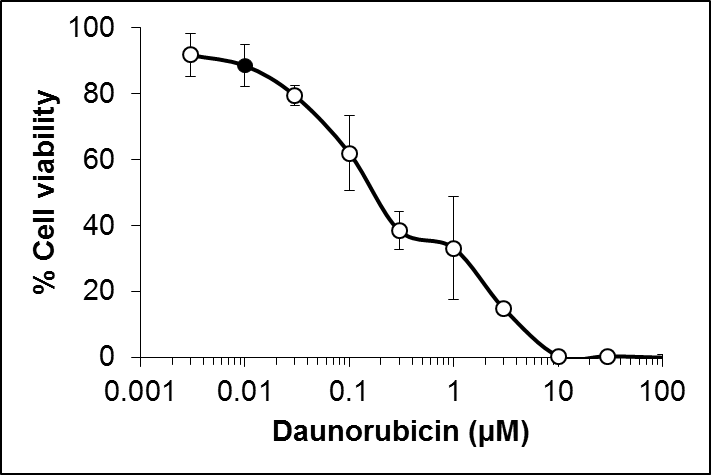

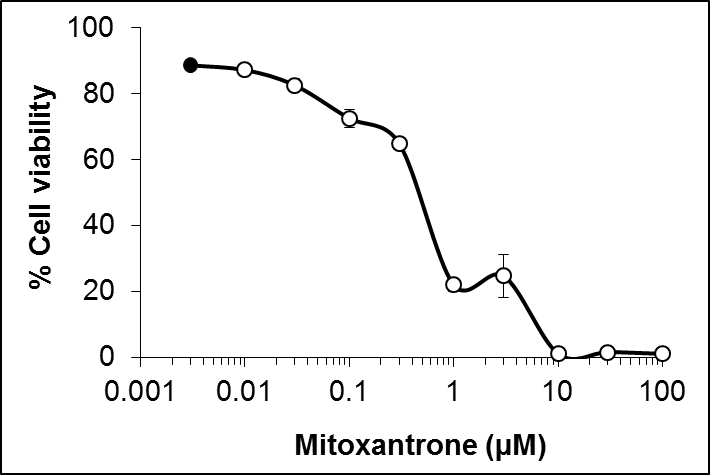


**Supplementary fig. S2:** The hiPSC-CMs response to 3 different doses of anthracyclines in terms of beating rate. The figures A, B and C show change in % beating rate after 48h drug exposure. Error bars represents mean ± Standard Deviation. Beating rate was determined using the xCELLigence RTCA cardio system at threshold 10.

**A: B. C.**


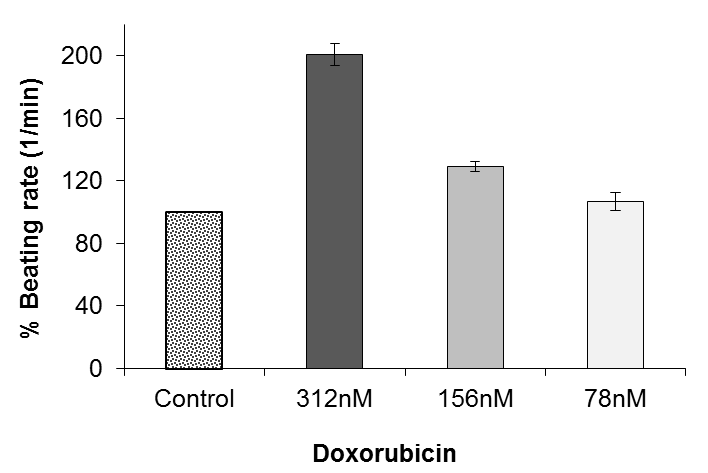

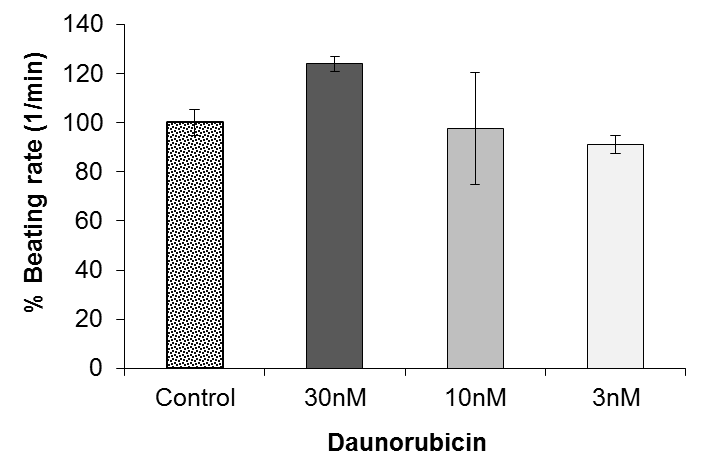

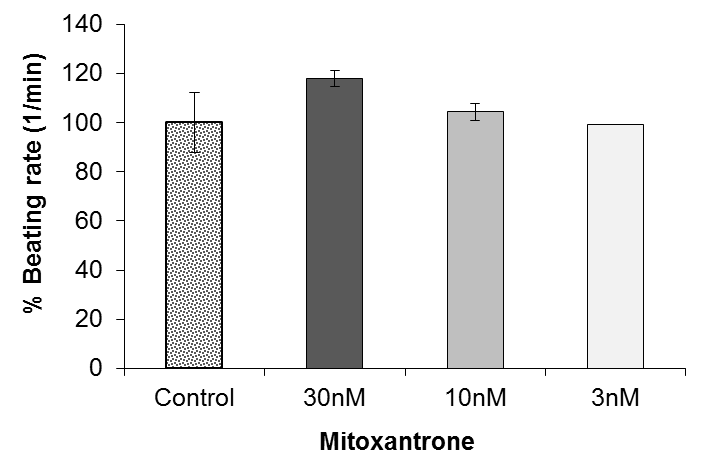


**Supplementary fig. S3:** Cardiomyocytes beating activity after anthracylines exposure. The representative 12 seconds beating traces captured in **A.** control, **B.** Daunorubicin treated and **C.** Mitoxantrone treated hiPSC-CMs after 48 h drug exposure. Beating profile shows no significant changes in beating activity.

1. **B. C.**


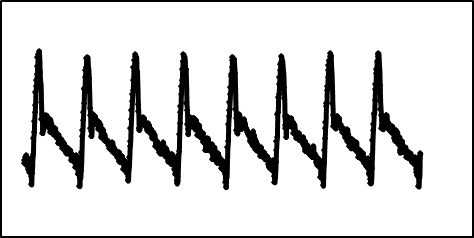

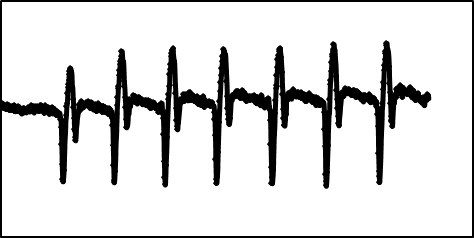

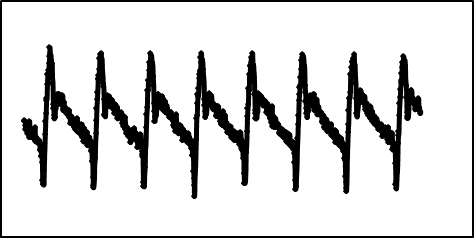


Mitoxantrone (3 nM)

Daunorubicin (10 nM)

Control

**Supplementary fig. S4:** Cell viability images of hiPSC-CMs after 48 h exposure of daunorubicin and mitoxantrone. Scale bar represents 50µm.

1. **B. C.**


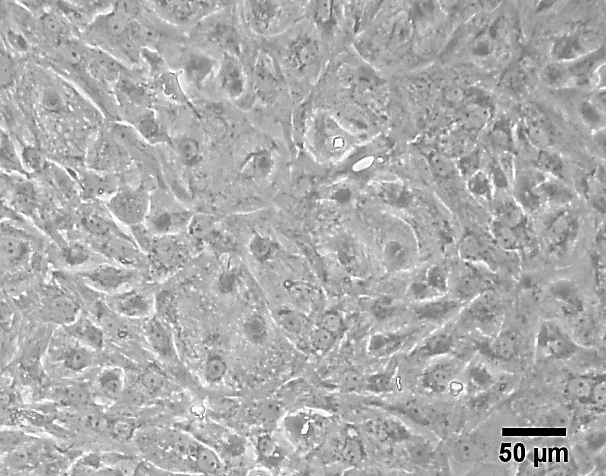

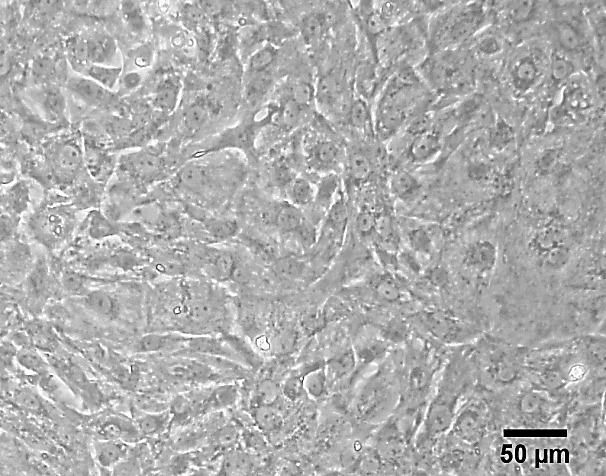
 **
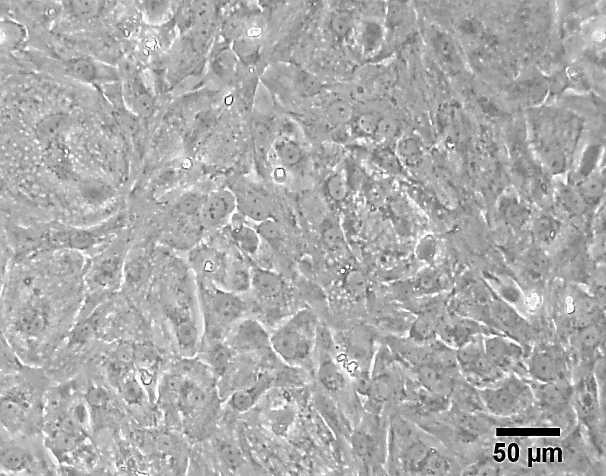
**

Mitoxantrone (3 nM)

Daunorubicin (10 nM)

Control

**Supplementary fig. S5:** Genes involved in the oxidative stress response found to be highly up-regulated in DOX-Day2 and DOX-Day6 cardiomyocytes (fold change relative to control).


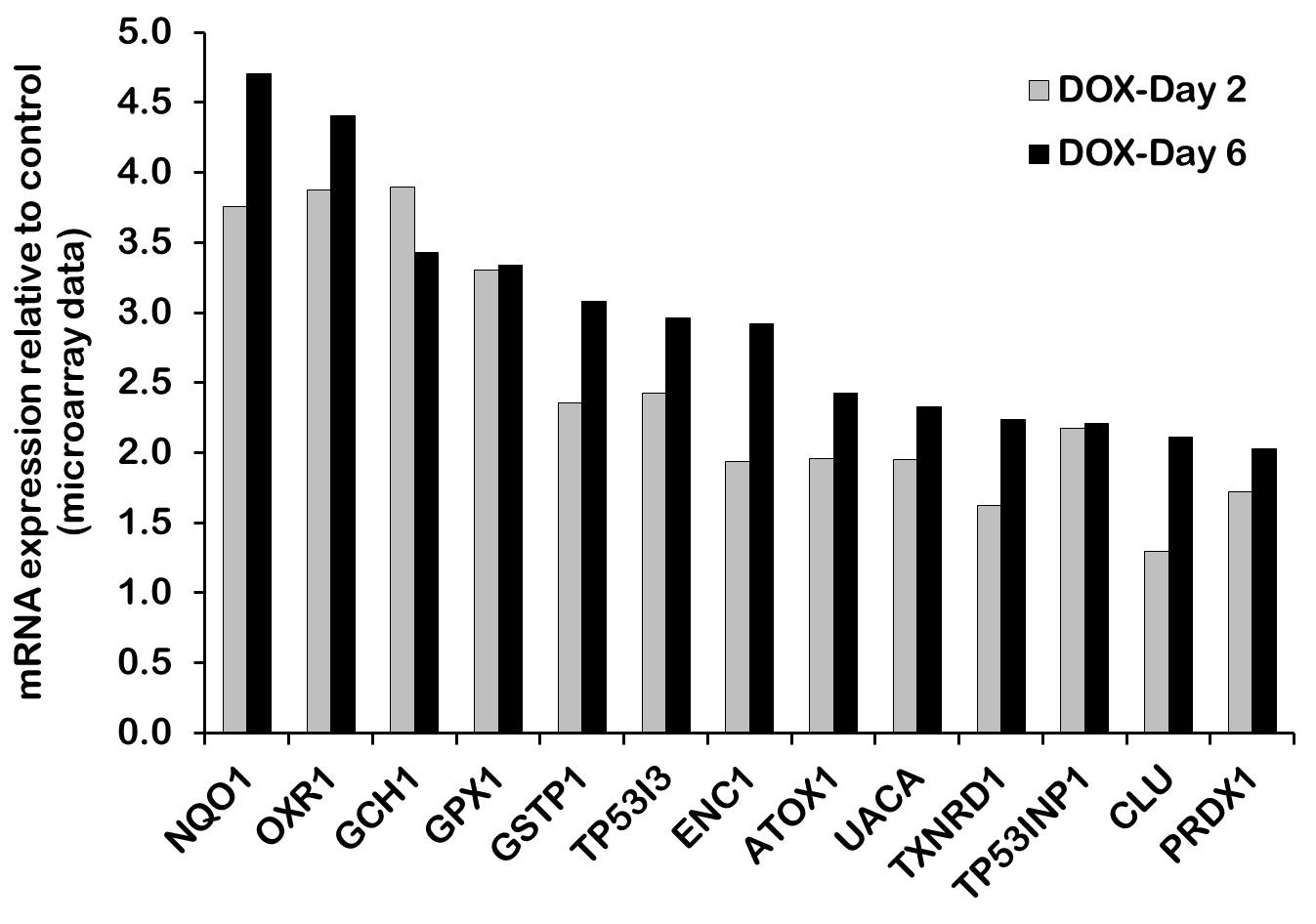

Supplement: Supplementary file 1 — Supplementary material 1 (DOCX 1485 kb) [file 204_2015_1623_MOESM1_ESM.docx]
